# Membrane Targeted Azobenzene Drives Optical Modulation of Bacterial Membrane Potential

**DOI:** 10.1002/advs.202205007

**Published:** 2023-01-29

**Authors:** Tailise Carolina de Souza‐Guerreiro, Gaia Bondelli, Iago Grobas, Stefano Donini, Valentina Sesti, Chiara Bertarelli, Guglielmo Lanzani, Munehiro Asally, Giuseppe Maria Paternò

**Affiliations:** ^1^ School of Life Sciences University of Warwick Coventry CV4 7AL UK; ^2^ Center for Nanoscience and Technology Istituto Italiano di Teconologia Milano 20133 Italy; ^3^ Physical and Theoretical Chemistry Laboratory Oxford OX1 3QZ UK; ^4^ Department of Chemistry, Materials and Chemical Engineering “Giulio Natta” Politecnico di Milano Milano 20133 Italy; ^5^ Department of Physics Politecnico di Milano Milano 20133 Italy

**Keywords:** bacterial cell electrophysiology, bacterial electrical signaling, bioelectricity, nanomaterials, optostimulation, photonics

## Abstract

Recent studies have shown that bacterial membrane potential is dynamic and plays signaling roles. Yet, little is still known about the mechanisms of membrane potential dynamics regulation—owing to a scarcity of appropriate research tools. Optical modulation of bacterial membrane potential could fill this gap and provide a new approach for studying and controlling bacterial physiology and electrical signaling. Here, the authors show that a membrane‐targeted azobenzene (*Ziapin2*) can be used to photo‐modulate the membrane potential in cells of the Gram‐positive bacterium *Bacillus subtilis*. It is found that upon exposure to blue–green light (*λ* = 470 nm), isomerization of *Ziapin2* in the bacteria membrane induces hyperpolarization of the potential. To investigate the origin of this phenomenon, ion‐channel‐deletion strains and ion channel blockers are examined. The authors found that in presence of the chloride channel blocker idanyloxyacetic acid‐94 (IAA‐94) or in absence of KtrAB potassium transporter, the hyperpolarization response is attenuated. These results reveal that the *Ziapin2* isomerization can induce ion channel opening in the bacterial membrane and suggest that *Ziapin2* can be used for studying and controlling bacterial electrical signaling. This new optical tool could contribute to better understand various microbial phenomena, such as biofilm electric signaling and antimicrobial resistance.

## Graphical abstract

Here, a membrane‐targeted azobenzene is employed to modulate optically the membrane potential of bacteria in a spatiotemporal precise manner. Non‐genetic optostimulation, which relies on the cross talking between light‐excited states and cell electrical status, can permit to gain precious insights into important microbial biological processes, such as bacterial electrophysiology, biofilm electric signaling, and antimicrobial resistance.

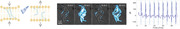

## 1 Introduction

Recent studies have revealed that bacterial membrane potential can exhibit neuron‐like spiking and oscillatory dynamics.^[^ ^1^, ^2^, ^3^ ^]^ For example, spiking membrane potential dynamics in *Escherichia coli* plays a role in mechanosensation.^[^ ^4^ ^]^ Oscillatory dynamics of *B. subtilis* coordinate glutamate metabolism^[^ ^2^, ^5^, ^6^ ^]^ and allows nutrient time‐sharing between colonies^[^ ^7^ ^]^ and multi‐species biofilm formation.^[^ ^8^ ^]^ Bacterial membrane potential is also tied to spore formation,^[^ ^9^ ^]^ germination,^[^ ^10^ ^]^ and cellular responses to ribosome‐targeting antibiotics.^[^ ^11^, ^12^ ^]^ Electrical stimulation can induce vitality‐dependent responses in bacteria^[^ ^13^, ^14^ ^]^ and cell type specific proliferation.^[^ ^15^ ^]^

In neurons and muscles, action potential is underpinned on a cascade of ion channel opening. External stimuli trigger a sequence of specific ionic conductance changes, which results in a finite temporal pattern of action potential. On the other hand, while several ion channels have been identified to mediate bacterial electrical signaling individually,^[^ ^1^, ^16^ ^]^ it is still unclear whether bacteria have such a cascade of ion channel signaling that responds to a transient external stimulus.

**Figure 1:**
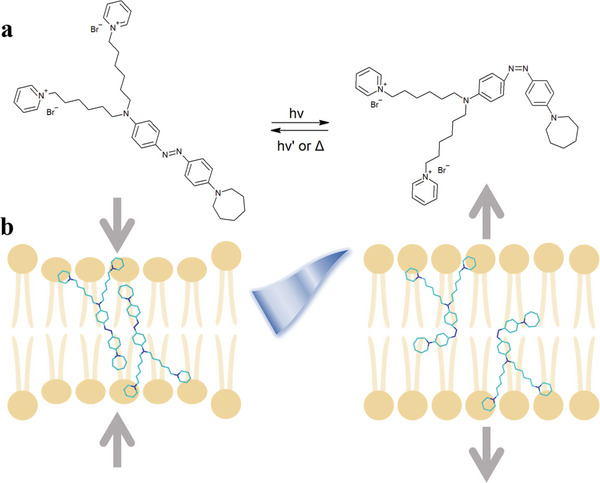
Illustrative diagram of photo‐induced *Ziapin2* isomerization. a) Molecular structure of *Ziapin2* and representation of its isomerization reaction. b) The optomechanical action of *Ziapin2* when sitting in the lipid membrane. In the *trans* elongated form, *Ziapin2* is able to dimerize within the lipid membrane, leading to a decrease in the thickness and an increase in the membrane capacitance. On the other side, illumination with cyan light (470 nm) triggers *Ziapin2* isomerization into its cis bent form, an effect that disrupts the dimers and leads to an increase in the thickness and a decrease of the membrane capacitance.^[^ ^22^, ^23^, ^24^, ^25^, ^26^ ^]^

Optostimulation technology permits to elicit and monitor signaling rapidly, remotely, and with high spatiotemporal precision, which can therefore be a useful tool for both basic and applied research into bacterial cell electrophysiology.^[^ ^17^ ^]^ In neuroscience, genetic and non‐genetic optomodulation techniques are increasingly recognized as a transformative technology.^[^ ^18^, ^19^, ^20^, ^21^ ^]^ Recently, we introduced a molecular optomechanical light transducer, named *Ziapin2*, which is able to drive optical modulation of the electrical properties of membranes in primary culture neurons and in vivo mouse brain.^[^ ^22^ ^]^ *Ziapin2* is an amphiphilic azobenzene with a strong non‐covalent affinity to the plasma membrane^[^ ^22^, ^23^ ^]^ (**Figure** **1**). Its optomodulation ability resides in the fact that the dark‐adapted *trans* isomer causes a thinning of the lipid bilayer via a dimerization mechanism, while illumination with visible light (≈470 nm) leads to a membrane relaxation that follows the disruption of the azobenzene dimers (Figure 1). In other words, the membrane thinning effect by *Ziapin2* is reversed upon light irradiation. Consequently, this brings about a light‐driven decrease of the membrane capacitance and causes transient hyperpolarization. Importantly, it was demonstrated that *Ziapin2* is nontoxic to neurons and can be used to activate cortical networks when injected into the mouse somatosensory cortex.^[^ ^22^ ^]^ The mechanism of action of *Ziapin2* optomodulation suggests that, in principle, it may be used to control the membrane potential of bacteria. However, this possibility is not trivial as the physicochemical environment of bacterial cell surface is significantly different from neurons.

In this study, we explored the possibility to extend the use of *Ziapin2* to bacteria and investigated if it triggers bioelectrical response. Using the Gram‐positive bacterium Bacillus *B. subtilis* as model organism, we demonstrate the optical modulation of bacterial membrane potential driven by visible light illumination. We show that *Ziapin2* associates with *B. subtilis* membrane and can trigger a hyperpolarization following optical stimulation. Intriguingly, the optomodulation experiments unveiled the involvement of KtrAB potassium transporter and uncharacterized chloride channels. These findings not only provide the proof of concept for the optical modulation of bacterial membrane potential using a photoswitching molecule but also suggest the existence of a bacterial bioelectric signaling that involves multiple ion channels.

## 2 Results

### 2.1 *Ziapin2* Associates with the Plasma Membrane in *B. Subtilis*

**Figure 2:**
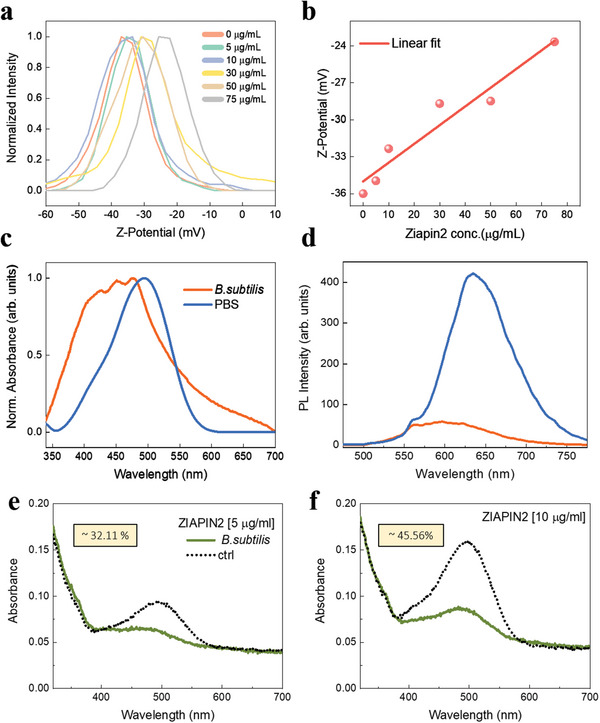
*Ziapin2* can associate with *B. subtilis* membrane. a) Variation of the distribution of *ζ* potential of *B. subtilis* cells as a function of *Ziapin2* concentration. b) Linear trend of *ζ* potential as a function of *Ziapin2* concentration. c) UV–vis and d) PL spectra of 10 µg mL^−1^ *Ziapin2* in PBS (blue lines) and in *B. subtilis* cells (orange lines). PL spectra were normalized to both lamp intensity and ground state absorption, to obtain a relative PL quantum yield among the two samples. Cellular uptake experiments performed for 0.5 and 10 µg mL^−1^ of *Ziapin2*, in the supernatant (dashed line) and in the cell fraction (continuous line). See Figure S1 (Supporting Information) for the comparison between dark and light (excitation at 470 nm) conditions.

To explore whether *Ziapin2* can be used to modulate bacterial membrane potential with light, we began by examining the association of *Ziapin2* with cells. *B. subtilis* cells were incubated with 5 and 10 µg mL^−1^ *Ziapin2* in dark and under 470‐nm light. First, we measured the *ζ* potential of cells by their electrophoretic mobility.^[^ ^27^, ^28^ ^]^ The *ζ* potential is the electrical potential at a colloid particle slipping plane, consisting in the interface separating mobile fluid from the fluid that remains attached to the particle surface. It is thus expected that when the positively charged *Ziapin2* is associated with the bacterial membrane, the overall negative surface potential of the cell should become less negative. Our measurements indeed show a linear rise in *ζ* potential with increasing *Ziapin2* concentrations, indicating the association of *Ziapin2* with the surface of *B. subtilis* cells (**Figure** **2**a,b).

Partitioning of *Ziapin2* into the bacterial membrane was further supported by UV–vis and photoluminescence spectroscopies, as it happens for eukaryotic cells.^[^ ^22^, ^25^ ^]^ Specifically, the absorption spectrum of *Ziapin2* in bacteria displays a better resolved vibronic progression and a broader linewidth in comparison to *Ziapin2* in phosphate buffer saline (PBS) (Figure 2c), an effect that has been attributed to H‐aggregation of the chromophore inside the lipid membrane and can be linked to *Ziapin2* dimerization at this location.^[^ ^23^, ^29^, ^30^ ^]^ Photoluminescence (PL) is more sensitive to the local environment than absorption as emission occurs after re‐equilibration within the solvent cage and, indeed, shows clear changes in both spectral position and relative emission quantum yield. In particular, in PBS we observe both an almost eightfold increase of the relative quantum yield and a marked redshift (40 nm) in comparison to *Ziapin2* PL in bacteria (Figure 2d). The enhanced and red‐shifted PL can be linked to the suppression of the isomerization ability in water owing to the formation of excimer aggregates, while the membrane environment protects *Ziapin2* isomerization. Since this is an efficient non‐radiative deactivation pathway,^[^ ^23^ ^]^ *Ziapin2* exhibits a relatively low emission when sitting in the membrane. Finally, the measurements of UV–vis absorption for cell fraction and supernatant showed that *B. subtilis* cells retain ≈32% and ≈45% of *Ziapin2* at 5 and 10 µg mL^−1^, respectively (Figure 2e,f). No significant difference was observed between dark and 470‐nm light conditions (Figure S1, Supporting Information). These results suggest that *Ziapin2* association is not affected by the isomerization reaction and, hence, the photoreaction may be used for altering the membrane capacitance by light.

### 2.2 *Ziapin2* Can Undergo Photo‐Isomerization in the Bacterial Membrane

**Figure 3:**
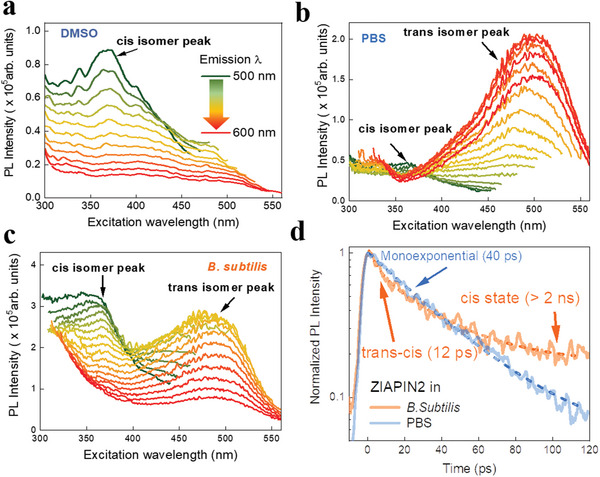
*Ziapin2* can undergo isomerization while in bacterial membrane. Excitation–emission profiles of *Ziapin2* (10 µg mL^−1^) in a) DMSO, b) PBS, and c) *B. subtilis* cells. For each curve in plots (a–c) the emission wavelength is fixed at a value between 500 and 600 nm, with 10 nm steps (color lines go from green to red passing from 500 to 600 nm). d) Time‐resolved PL decay curves of *Ziapin2* in PBS (orange line) and *B. subtilis* cells (blue line). The dashed lines represent the exponential best‐fit for the two curves.

To test whether *Ziapin2* can undergo light‐induced isomerization while embedded in the bacterial membrane, we employed both steady state and time‐resolved photoluminescence spectroscopy. In particular, we acquired excitation/emission maps to reconstruct the *Ziapin2* deactivation scenario upon photoexcitation. The Vavilov–Kasha rule is fulfilled when the excitation profile and the absorption spectrum overlap; after absorption, the molecule relaxes to the lower excited state before emission occurs. If the two curves have different shapes, it indicates that the branching ratio between radiative and non‐radiative decay paths varies with wavelength. As a test bench, we collected the PL excitation profile in DMSO, which is the solvent of choice for *Ziapin2*. Here, we observed the signature of emission from the *cis* isomer, namely an excitation peak at 370 nm (**Figure** **3**a), and a relatively smaller peak at around 500 nm that accounts for the excitation of *trans* isomer.^[^ ^23^ ^]^ The ratio between the two excitation peaks can be related to the composition of the photostationary state, which in DMSO is *cis*‐enriched (≈70%) as it has been also observed in our previous studies.^[^ ^23^, ^26^ ^]^ The *cis* isomer peak, on the other hand, was barely visible in PBS (Figure 3b), with the *trans* conformer peak at 500 nm taking precedence. This result implies that the isomerization of *Ziapin2* in PBS is hampered, resulting in a radiative deactivation within the *trans* manifold. Intriguingly, both the *cis* and *trans* isomer peaks coexisted in *B. subtilis* suspension (Figure 3c) with a comparable peak intensity (photostationary state composition ≈50% *cis*).^[^ ^26^ ^]^ This suggests that the bacterial membrane's physicochemical environment restores at least partially the isomerization ability of *Ziapin2*. We also carried out time‐resolved PL experiments (Figure 3d). While the decay in PBS was mono‐exponential (*τ*_1_ = 40 ps), the decay in *B. subtilis* cells was bi‐exponential with the first component lifetime (*τ*_1_ ≈ 12 ps), consistent with *Ziapin2* isomerization in artificial and natural membranes.^[^ ^22^, ^23^ ^]^ All together, these data provide strong evidence for *Ziapin2* isomerization in the bacterial membrane.

### 2.3 Light Induces a Transient Hyperpolarization in *Ziapin2*‐Treated Bacteria

Given these results, we examined the capability of *Ziapin2* to evoke membrane potential dynamics in bacterial cells.^[^ ^22^ ^]^ This would be the first translation of our non‐genetic optical stimulation approach into the prokaryotic realm. First, we evaluated the cell viability upon administration of *Ziapin2* via plate reader assay, which showed that *Ziapin2* has no significant effect on cell growth when used at <2.5 µg mL^−1^ (Figure S2, Supporting Information). Then we proceed to study bacterial membrane potential by epifluorescence time‐lapse microscopy using an optical probe, Tetramethyl rhodamine methyl ester (TMRM). TMRM is a lipophilic cationic dye that accumulates in cells with more negative membrane^[^ ^31^ ^]^ and has a fast equilibration time, <1 min (Figure S3, Supporting Information). The fluorescence measurements were used to calculate the membrane potential change (Δ*V*_m_) from the resting potential (see Experimental Section). According to the mechanism of action of *Ziapin2*, its *trans* form thins the membrane and hence increases the capacitance, which then depolarizes the membrane (Figure 1). To test if such a depolarization by *Ziapin2* also occurs in bacteria, we measured the resting membrane potential levels with and without *Ziapin2*. The result showed that *Ziapin2* indeed depolarizes the plasma membrane in the absence of 470‐nm light stimulation (Figure S4, Supporting Information).

**Figure 4:**
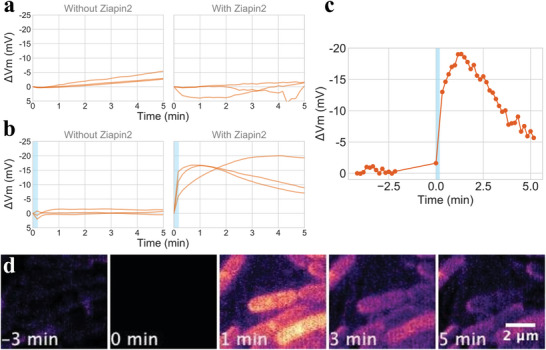
*Ziapin2* modulation of *B. subtilis* membrane potential depends on 470 nm light stimulation. a–c) Membrane potential change (Δ*V*_m_) over time, measured by TMRM fluorescence. See Experimental Section regarding the conversion of TMRM fluorescence into millivolt. Three biological repeats are plotted for panels (a) and (b). The origin of time was chosen as immediately before light stimulation. The fluorescence at time 0 was used as the resting potential. Mean trace; a) without light stimulation without (left) and with (right) *Ziapin2*; b) with 10 s light stimulation (light blue) without (left) and with (right) *Ziapin2*. Each trace shows the results from three independent experiments. Average number of cells analyzed per experiment repeat for each condition are: without Ziapin2, no light stimulation: 1300; with Ziapin2, no light stimulation: 2200; without Ziapin2, with light stimulation: 1800; with Ziapin2, with light stimulation: 500. Blue horizontal box indicates the timing and duration of 470‐nm light stimulation (20 mW mm^−2^). c) Representative single‐cell time‐trace of Ziapin‐induced membrane potential dynamics before and after 470 nm light stimulation. d) Film‐strip images of TMRM signal with cells with *Ziapin2*. Cells were stimulated for 10 s by light immediately after at time 0.

To examine if photo‐induced isomerization of *Ziapin2* can cause a transient change in membrane potential, we performed time‐lapse microscopy where cells were stimulated by 470 nm light for 10 s in presence of *Ziapin2*. In the absence of 470‐nm light stimulation (negative control), TMRM signal was stable over the course of our time‐lapse experiment, regardless of the presence or the absence of *Ziapin2* (**Figure** **4**a and Figure S5, Supporting Information). We also confirmed that a 470‐nm light stimulation does not cause a significant change in TMRM signal when *Ziapin2* is not present (Figure 4b, left). In the presence of *Ziapin2*, we observed a rise in TMRM signal following light stimulation, suggesting a hyperpolarization by ≈15 mV (Figure 4b, right; also see Movie S1, Supporting Information). Figure 4c illustrates the TMRM dynamics of a representative cell before and after light stimulation. The mean TMRM signal is stable before photo stimulation, which then undergo a photo‐induced hyperpolarization followed by a gradual rebound (Figure 4c). Varying the intensities of 470‐nm light, we found that the light intensity >2 mW mm^−2^ could be sufficient to cause a hyperpolarization response (Figure S6, Supporting Information). These results demonstrate, for the first time, that a photo‐switch *Ziapin2* can indeed be used to modulate the bacterial membrane potential using light.

**Figure 5:**
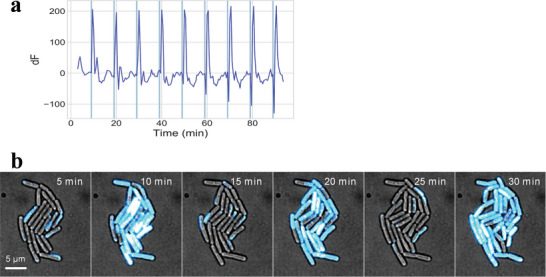
Periodic photo‐induced hyperpolarization. a,b) Cells were cultured with Ziapin2 and stimulated by 470 nm light for 10 s every 10 min. Membrane potential was measured using TMRM. The change in TMRM fluorescence (dF) over time from a representative microcolony. Please also see the Movies S1 and S2 (Supporting Information).

To examine whether the photo‐induced hyperpolarizations can be repeated, we conducted a 100‐min time‐lapse microscopy experiment where cells are stimulated by light every 10 min. The stimulation was kept the same as the experiment in Figure 4. The result showed that a periodic hyperpolarization in a growing culture of cells (**Figure** **5** and Movie S2, Supporting Information). This result clearly demonstrates the repeatability of the light‐induced hyperpolarization.

### 2.4 Light‐Induced *Ziapin2* Isomerization Leads to the Opening of Potassium and Chloride Channels

The photo‐induced hyperpolarization in bacterial cells lasted for several minutes (Figure 4). This dynamics is much slower than *Ziapin2* single isomerization event which occurs in the picosecond time regime and reaches a cis‐enriched photostationary state within ≈20 s, while the *cis*→*trans* relaxation usually happens in less than 1 min.^[^ ^22^, ^23^, ^24^ ^]^ This discrepancy in time scale could be accounted for by a slower bioelectrical response that is triggered by *Ziapin2* isomerization. More specifically, we hypothesized that *Ziapin2* isomerization triggers opening of ion channels on bacterial membrane, which results in a transient hyperpolarization.

**Figure 6:**
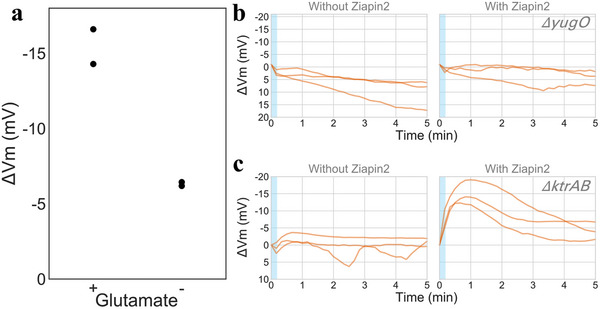
Photo‐induced hyperpolarization response depends on glutamate and KtrA‐KtraB potassium transporter a) Glutamate is important for the extent of *Ziapin2* modulation of membrane potential dynamics. The peak hyperpolarization response to light in the media with and without glutamate. Data from two independent experiments. Each dot is average of >100 cells. Membrane potential change following light stimulation (blue) with b) *yugO* and c) *ktrAB* deletion strains. *yugO* does not impact the hyperpolarization observed upon light stimulation. KtrA‐KtraB potassium channel is involved in *Ziapin2*‐induced membrane potential modulation, as its deletion eliminates the hyperpolarization observed upon exposure to 470 nm light. Each trace shows the results from three independent experiments. Average number of cells analyzed per experiment repeat for each condition are: *yugO*‐ without Ziapin2, with light stimulation: 250; with Ziapin2, with light stimulation: 230; *ktrAB*‐ without Ziapin2, with light stimulation: 205; with Ziapin2, with light stimulation: 129.

If the light‐induced hyperpolarization is a result of biological ion channel dynamics, one would expect the response dynamics depends on the culture conditions, in particular the ones that impact the opening of ion channels. To this end, we focused on glutamate because it is known to play a central role in biofilm electrical signaling by gating the YugO potassium channel.^[^ ^2^, ^5^ ^]^ Cells were cultured in the media with and without glutamate and examined by time‐lapse fluorescence microscopy. This experiment showed that light stimulation causes a weaker hyperpolarization response with cells in the media without glutamate (**Figure** **6**a). This data supports the hypothesis that the photoinduced membrane potential dynamics involves a biological process.

Toward better understanding the biological machineries of the process, we utilized potassium channel deletion mutant strains. We first tested the *yugO* deletion strain because the potassium channel encoded by this gene is known to mediate biofilm electrical signaling.^[^ ^2^ ^]^ YugO channel is structurally similar to the classic KcsA potassium channel with a TVGYG selectivity filter motif (Figure S7, Supporting Information). The photo‐stimulation microscopy experiment was conducted in the same way as the wild type. We first confirmed that the TMRM signal is stable over the course of our experiment without *Ziapin2*. With *Ziapin2*, the TMRM signal underwent a transient signal increase upon light stimulation, similar to the wild type (Figure 6b, see also Figure S8, Supporting Information for negative controls). These results suggest that YugO channel is dispensable for the light‐triggered hyperpolarization, in spite of its role in biofilm electrical signaling.

We next tested the mutant strain that lacks the genes encoding the high‐affinity potassium channel KtrAB, which belongs to TrK/Ktr/HKT super family.^[^ ^32^ ^]^ This potassium channel is involved in K+ uptake and it lacks the highly conserved TVGYG selectivity filter motif sequence. Due to its unique structure and electrophysiological property, this channel has been called as a “unusual K+ channel^[^ ^33^ ^]^”. The TMRM signal was less stable with this strain than the wildtype and showed gradual signal decay in our negative control experiments (Figure 6c, left panel). Upon exposure to 470 nm light, no significant change in membrane potential was observed (Figure 6c, right panel, and Figure S9a, Supporting Information). These results suggest that KtrAB potassium channel may alter the resting state and or play a role in the response dynamics.

**Figure 7:**
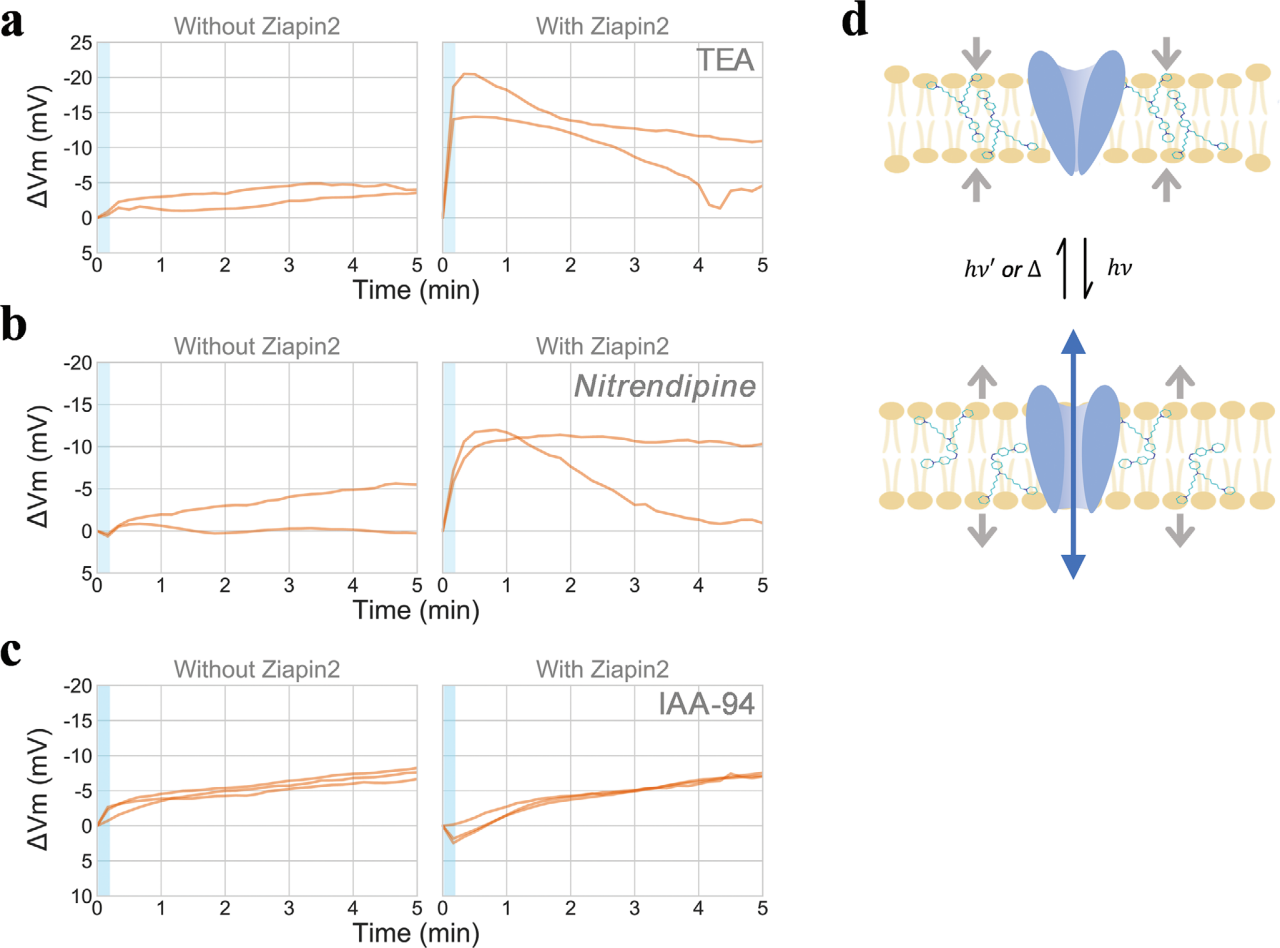
Chloride channel blocker attenuate the hyperpolarization response. Membrane potential change over time in the presence of ion channel blockers, a) the potassium blocker TEA, b) the calcium blocker Nitrendipine, and c) the chloride blocker IAA‐94. IAA‐94 impairs the hyperpolarization induced by *Ziapin2* upon light stimulation, suggesting chloride channels are involved in *Ziapin2*‐induced membrane potential dynamics. Mean behavior from independent experiments are shown in separate lines. Average number of cells analyzed per experiment: TEA—without Ziapin2, with light stimulation: 166; with Ziapin2, with light stimulation: 128; Nitrendipine‐ without Ziapin2, with light stimulation: 317; with Ziapin2, with light stimulation: 172. IAA‐94‐ without Ziapin2, with light stimulation: 209; with Ziapin2, with light stimulation: 290. d) Illustrative diagram showing the hypothetical model that photo‐induced *Ziapin2* isomerization causes opening of potassium and chloride ion channels.

Our understanding of *B. subtilis* ion channels is far from complete because the bacterial electrophysiology and bacterial electrical signaling are only recently gaining attention. Therefore, it is very likely that *Ziapin2* isomerization triggers opening of uncharacterized ion channels. To explore this possibility, we employed three ion channels blockers: namely, the potassium channel blocker tetraethylammonium (TEA), the calcium channel blocker Nitrendipine, and the chloride channel blocker Indanyloxyacetic acid‐94 (IAA‐94). It is worth noting that TEA blocks the entry region of potassium channel near c selective filter. However, it is unlikely to block KtrAB channel because of its structure and the sequence lacking TVGYG selective filter. The wildtype cells were treated with an ion‐channel blocker for 1 h before being used for photo‐stimulation microscopy experiments. The results showed that, in the presence of *Ziapin2*, cells treated with TEA or nitrendipine showed a TMRM signal increase upon light exposure, as it would happen in the absence of blockers (**Figure** **7**a,b). On the other hand, cells treated with IAA‐94 did not show a transient signal rise upon light stimulation (Figure 7c and Figure S9b, Supporting Information). Instead, we observed a slow gradual hyperpolarization which is likely unrelated to *Ziapin2* isomerization as the condition without *Ziapin2* showed a similar pattern. Altogether, our results suggest that *Ziapin2* isomerization causes gating of ion channels (Figure 7d). In other words, separate to biofilm electrical signaling, which is mediated by YugO, bacterial membrane is equipped with a machinery that can produce a bioelectric response to a fast voltage change by *Ziapin2* isomerization.

## 3 Discussion

We demonstrate that the membrane potential of *B. subtilis* can be controlled by light stimulation without genetic modifications. To the best of our knowledge, this is the first example of inducing membrane potential dynamics using light. We employed a membrane‐targeted azobenzene molecule, *Ziapin2*, which is able to drive modulation of the membrane capacitance and potential via an optomechanical effect. Under visible light illumination (*λ* ≈ 470 nm), we observed a transient hyperpolarization followed by a depolarization rebound. The time‐scale discrepancy between the relatively fast isomerization process and the long‐lasting biological effects prompted us to study the possible involvement of voltage‐gated ion channels. Intriguingly, we found that the potential modulation brought about by *Ziapin2* isomerization triggers the opening of the chloride channel, whose role is still largely uncharacterized for prokaryotes. More in general, this finding suggests that bacteria are equipped with bioelectric machinery that can respond to a fast voltage change. It is anticipated that future studies will further characterize the ion channels.

An important future research topic is elucidating the molecular mechanism of the bioelectric circuit. While cells exposed to the potassium channel blocker TEA exhibited photo‐stimulated membrane potential dynamics, *ktrAB* deletion strain did not show such a response. The blockage by TEA depends on an aromatic residue on the extracellular side of the channel,^[^ ^34^ ^]^ hence, it is possible that TEA does not block KtrAB channel. In a future project, we would also like to characterize the molecular identity of ion channels that are blocked by IAA‐94. While many bacteria carry genes encoding chloride channels, which are commonly used as the model for neural ion channels, the physiological roles of chloride channels are still largely elusive. We also note that, to the best of our knowledge, no chloride channels have been characterized in *B. subtilis*. Our finding could be a ground to elucidate the physiological roles of chloride channels. Another important group of channels to investigate further is mechanosensitive channels.^[^ ^35^ ^]^

To date, the bioelectronics community's efforts to interrogate cells have primarily been devoted to eukaryotes,^[^ ^36^, ^37^, ^38^ ^]^ yet the community has recently steered to the development of new interfaces for studying and controlling bacterial functions.^[^ ^1^, ^17^, ^39^, ^40^, ^41^ ^]^ The interest is mostly driven by the recent observation of neuron‐like electrical patterns, such as spiking^[^ ^3^ ^]^ and oscillation.^[^ ^2^, ^42^ ^]^ It is intriguing to analogously consider these signaling and circuits as forming a “bacterial brain” that regulates metabolism and adaptation/responsivity to external stimulus and stressors, such as drugs and antibiotics. The fact that the bacterial membrane potential can be dynamically controlled by external stimuli opens new and exciting opportunities to gain new biological insights connected to signaling roles of the bacterial membrane potential. Exogenous light stimulation is perfectly suited to serve to this role, as it permits to elicit signaling with high spatiotemporal precision and remotely, therefore surpassing some intrinsic limitation of electrode‐based methods, such as the need for contacting small, motile and highly heterogeneous bacterial cells.^[^ ^43^ ^]^

For these reasons, non‐genetic optostimulation has the potential to boost research in the field of bacterial electrophysiology, for instance via the use of patterned optical excitation/probing at different nodes of the neuron‐like network, as well as to facilitate the development of new synthetic‐biology technologies for the bioelectrical engineering of bacterial functions.

## 4 Experimental Section

### Synthesis of Ziapin2

*Ziapin2* has been synthesized according to the procedure that has been already published.^[^ ^22^, ^23^ ^]^ Unless otherwise stated, all chemicals and solvent were commercially available and used without further purification. Reactions of air‐ and water‐sensitive reagents and intermediates were carried out in dried glassware and under argon atmosphere. If necessary, solvents were dried by means of conventional method and stored under argon. Thin layer chromatography (TLC) was performed by using silica gel on aluminum foil, Sigma‐Aldrich). NMR spectra were collected with a Bruker ARX400. Mass spectroscopy was carried out with a Bruker Esquire 3000 plus.

### Growth Conditions and Preparation of Agarose Pads

Glycerol stock of *Bacillus subtilis* NCIB 3610 wild‐type strain (WT) was streaked on lysogeny‐broth (LB) 1.5% agar and incubated overnight in a 37 °C non‐shaking incubator. A single colony was picked from this plate, inoculated in LB and incubated at 37 °C shaking overnight. When specified in the text, a genetically modified strain (listed in Table S1, Supporting Information) was used instead of WT. When culturing a strain with antibiotic‐resistance genes, appropriate antibiotics were added to the media in the following concentrations: spectinomycin 100 µg mL^−1^; kanamycin 5 µg mL^−1^. Following overnight cultivation in liquid LB, cells were pelleted and washed once with resuspension media (RM)^[^ ^44^ ^]^ (RM; composition per1 liter: 46 µg FeCl_2_, 4.8 g MgSO_4_, 12.6 mg MnCl_2_, 535 mg NH_4_Cl, 106 mg Na_2_SO_4_, 68 mg KH_2_PO_4_, 96.5 mg NH_4_NO_3_, 219 mg CaCl_2_, 2 g monosodium L‐glutamate), and then incubated in RM at 37 °C shaking for an hour prior to microscopy assay. When specified in the text, glutamate was omitted from RM. Following incubation with RM, cells were then deposited on RM 1.5% weight/volume Low Melting Point (LMP) agarose pads prepared as described previously.^[^ ^9^, ^13^, ^14^ ^]^ When specified, TMRM, *Ziapin2* and ion channel blockers were added at the following concentrations: TMRM at 100 nm (Molecular Probes); *Ziapin2* at 1 µg mL^−1^; TEA (Sigma‐Aldrich) at 25 mm; Nitrendipine (Sigma‐Aldrich) at 10 µm; IAA‐94 (ApexBio Technology) at 100 µm.

### Time‐Lapse Microscopy and Light Stimulation

For time‐lapse and 470 nm light stimulation experiments, the fluorescence microscope Leica DMi8, equipped with an automated stage, Hamamatsu Orca‐flash 4.0 scientific CMOS (complementary metal–oxide–semiconductor) camera, a PeCon incubation system, and an objective lens HCX PL FLUOTAR 100x/1.30 OIL PH3, was used. TMRM fluorescence was detected with 500 ms exposure with Ex554/23 and Em609/54 filters (Semrock). The white LED of SOLA‐SM II light engine (Lumencor) was used with the power level 10/255 (≈4% of full power). For 470 nm stimulation Ex466/40 filter (Semrock) was used with 10 s exposure, and when specified in the text, the power level of the white LED of SOLA‐SM II light engine was varied from 2/255 to 10/255. The light power of the 470 nm stimulation was measured with the PM16‐121 power meter (Thorlabs) and the power density calculated in accordance with the area of the field of view.

Time‐lapse duration was 2 min before 470 nm stimulation, with acquisition interval of 10 s. Immediately after, another 5 min time‐lapse with same acquisition interval was conducted, where 470 nm exposure occurred once after the first TMRM image acquisition.

For TMRM equilibration time, cells were immobilized into microscopy glass bottom well slides (ibidi—µ‐Slide 8 Well) coated with poly‐L‐lysine (Sigma). Time lapse was performed for 20 min with 10 s interval for acquisition of TMRM fluorescence and bright field images.

### Membrane Potential Estimation

Estimation of *B. subtilis* membrane potential changes (Δ*V*_m_) from the fluoresce intensity was performed as described by Ehrenberg et al.^[^ ^31^ ^]^ using the following equation:

(1) $$\Delta V_{m}=\;V_{m}-V_{m,0}=\;-\frac{RT}{zF}\ln\left(\frac{\left(\mathrm{mpx}-I_{\mathrm{si}}\right)-R_{\mathrm{dex}}\left(I_{o}-I_{\mathrm{so}}\right)}{\left({\mathrm{mpx}}_{0}-I_{\mathrm{si}}\right)-R_{\mathrm{dex}}\left(I_{o}-I_{\mathrm{so}}\right)}\right)$$

where *V*_m_ is membrane potential, *V*_m,0_ is the resting membrane potential, *R* is the gas constant, *T* is the temperature in Kelvin, *z* is the charge of the dye, *F* is the Faraday constant, mpx is the mean pixel intensity from analyzed cells, mpx_0_ is the mean pixel intensity of cells before light stimulation, *I*_o_ is the mean background intensity, *I*_si_ is the autofluorescence of the cell (measured from cells without TMRM) and *I*_so_ is the background autofluorescence in the absence of TMRM. *R*_dex_ accounts for off‐focus signal. For the experimental setup, *R*_dex_ was determined to be 0.976 by taking the ratio of off‐focus and in‐focus image with rhodamine dextran as described by Ehrenberg et al..^[^ ^31^ ^]^ Calculations were performed with JupyterLab 1.2.6.^[^ ^45^ ^]^

### Steady‐Stated UV–vis/PL Spectroscopy and *ζ* Potential Measurements

Cells were suspended in PBS to OD_600nm_ = 0.5. For *ζ* potential measurements, 100 mL of each sample was diluted into 900 mL PBS. The measurements were performed on a Malvern Zetasizer Nano ZS (Malvern Instruments, Malvern, U.K.) at RT. Data points given are an average of 3 biological replicates with 3 measurements each.

UV–vis absorption measurements were performed using a Perkin Elmer Lambda 1050 spectrophotometer, with deuterium (180–320 nm) and tungsten (320–3300 nm) lamps, a monochromator and three detectors (photomultiplier 180–860 nm, InGaAs 860–1300 nm, and PbS 1300–3300 nm). Absorption spectra were normalized according to a reference spectrum taken at 100% transmission (without the sample), 0% transmission (with an internal shutter), and in the presence of the reference solvent. For the PL measurements and the excitation profiles an iHR320Horiba NanoLog Fluorometer was employed, equipped with a Xenon lamp, two monochromators, and two detectors (photomultiplier and InGaAs).

### *Ziapin2* Cellular Uptake Experiments

Cells suspended in PBS were stained with different concentrations of *Ziapin2* and kept at 37 °C for 60 min in dark. The samples were then centrifuged and 200 µL of each supernatant was transferred to a clean 96‐well plate for UV–vis absorption with a Tecan Spark10m plate reader. The light excited samples (LED 470 nm) were treated using the following illumination protocol: 10 min of light followed by 10 min in dark conditions, repeated three times. Absorbance was measured at 490 nm. Control samples with no cells were treated the same, and their absorbance values represented the total molecule for reference. All conditions and controls were measured in triplicate.

### Time‐Resolved PL Measurements

TRPL experiments were carried out using a femtosecond laser source coupled to a streak camera detection system (Hamamatsu C5680). A Ti:sapphire laser (Coherent Chameleon Ultra II, pulse bandwidths of B140 fs, repetition rate of 80 MHz, and maximum pulse energy of 50 nJ) was used to pump a second‐ harmonic crystal (b‐barium borate) to tune the pump wavelength to 470 nm. The measurements here shown were performed recording the first 130 ps of decays, with an IRF of 4.1 ps. When required, a Peltier cell was used in order to control the temperature of the sample.

## Conflict of Interest

The authors declare no conflict of interest.

## Supporting information

Supporting Information

Movie S1

Movie S2

## Data Availability

The data that support the findings of this study are available from the corresponding author upon reasonable request.
