# Supplementary material for: Membrane Targeted Azobenzene Drives Optical Modulation of Bacterial Membrane Potential
Source: Adv Sci (Weinh). 2023 Jan 29;10(8):2205007. doi: 10.1002/advs.202205007 (PMC10015841; doi:10.1002/advs.202205007)
Supplement: Supplementary file 1 — Supporting Information [file ADVS-10-2205007-s002.pdf]

Supplementary information for

Membrane Targeted Azobenzene Drives Optical Modulation of Bacterial Membrane Potential

Tailise Souza[1#], Gaia Bondelli[2#], Iago Grobas[3], Stefano Donini[2], Valentina Sesti[4], Chiara Bertarelli[4], Guglielmo Lanzani[2,5], Munehiro Asally[1*] & Giuseppe Maria Paternò[2,5*]

[1]School of Life Sciences, University of Warwick, Coventry, CV4 7AL, UK
[2]Center for Nanoscience and Technology, Istituto Italiano di Teconologia, Milano, 20133, Italy
[3]Physical and Theoretical Chemistry Laboratory, Oxford, OX1 3QZ, United Kingdom
[5]Department of Chemistry. Materials and Chemical Engineering "Giulio Natta" Politecnico di Milano, Milano, 20133, Italy
[5]Department of Physics, Politecnico di Milano, Milano, 20133, Italy

#These authors contributed equally to this work
*Correspondence to be addressed to Munehiro Asally (m.asally@warwick.ac.uk) and Giuseppe Maria Paternò (giuseppemaria.paterno@polimi.it)

Supplementary data

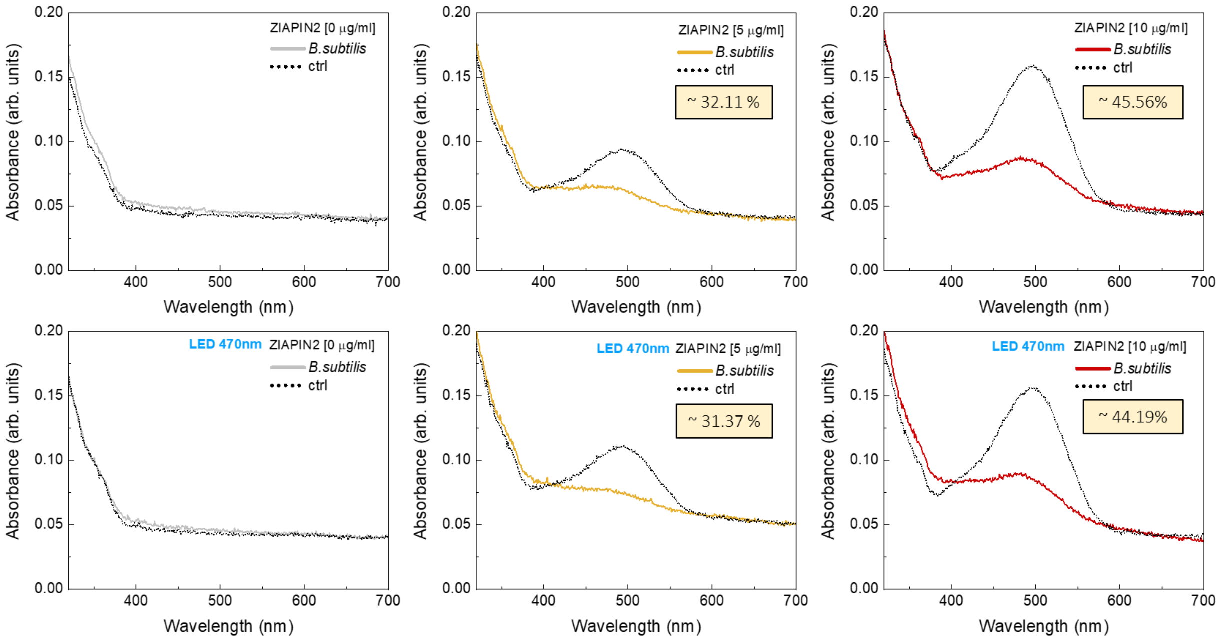

Figure S1. Cellular uptake experiments performed for 0, 5 and 10 µg/mL of Ziapin2, in the supernatant (dashed line) and in the cell fraction (continuous line). We carried out the experiment in dark and under illumination (470 nm), showing that light excitation does not cause ZIAPIN2 detachment from cells

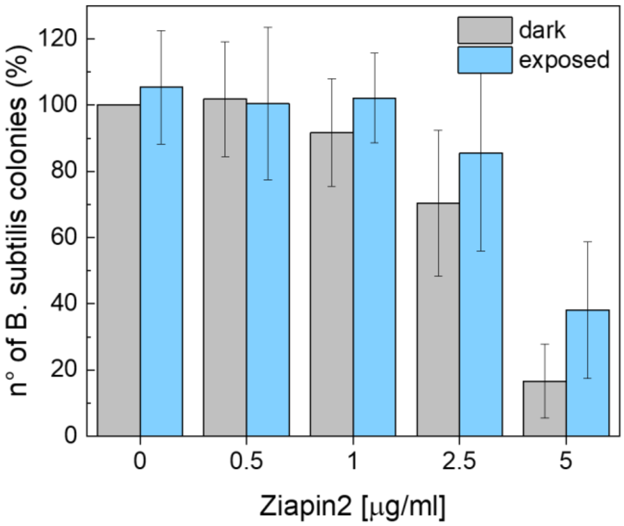

Figure S2. *B. subtilis* cells viability upon administration of Ziapin2 assessed via plate reader assay under dark and light (470 nm) conditions.

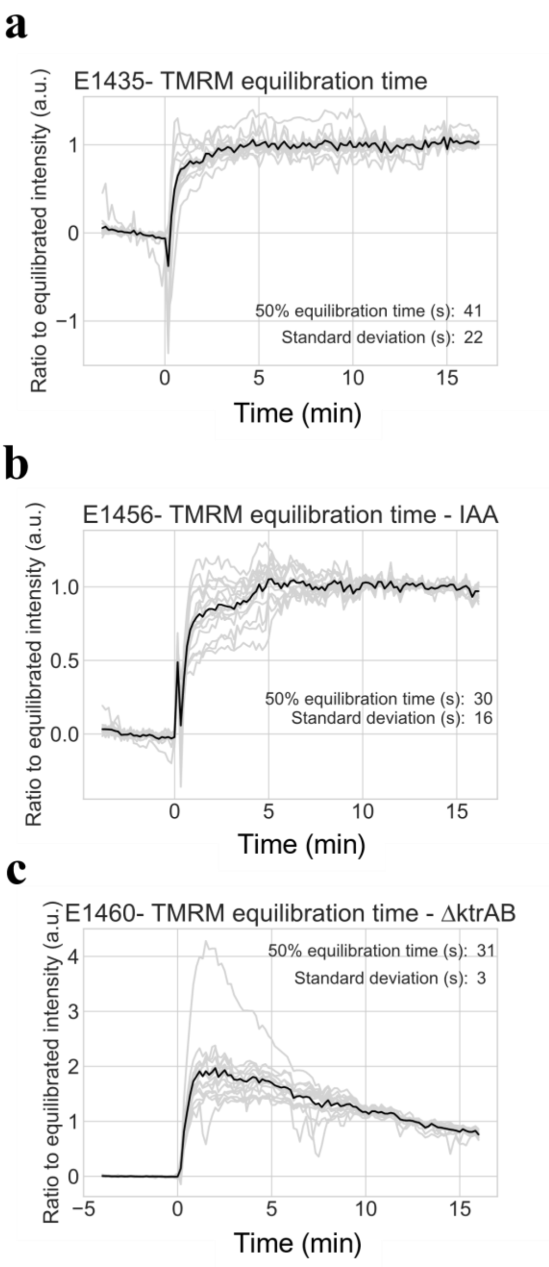

Figure S3. a) Upon addition of TMRM, WT cells achieved 50% of the maximum intensity in less than a minute. b) Cells exposed to IAA-94 showed a similar equilibration time, c) as did Δ*ktrAB.*

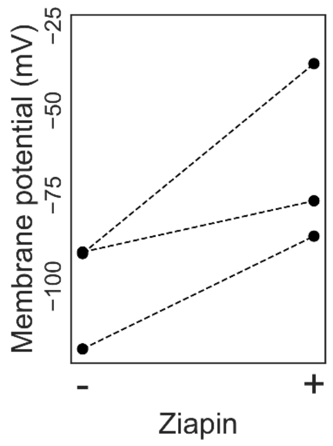

Figure S4 – *Ziapin2* depolarizes bacterial resting membrane potential – The resting membrane potential of Bacillus subtilis is depolarized upon exposure to Ziapin2 due its association to the membrane and consequent thinning due to the trans conformation and dimerization. Black dots are the average measurements of multiple cells, from 3 biological repeats.

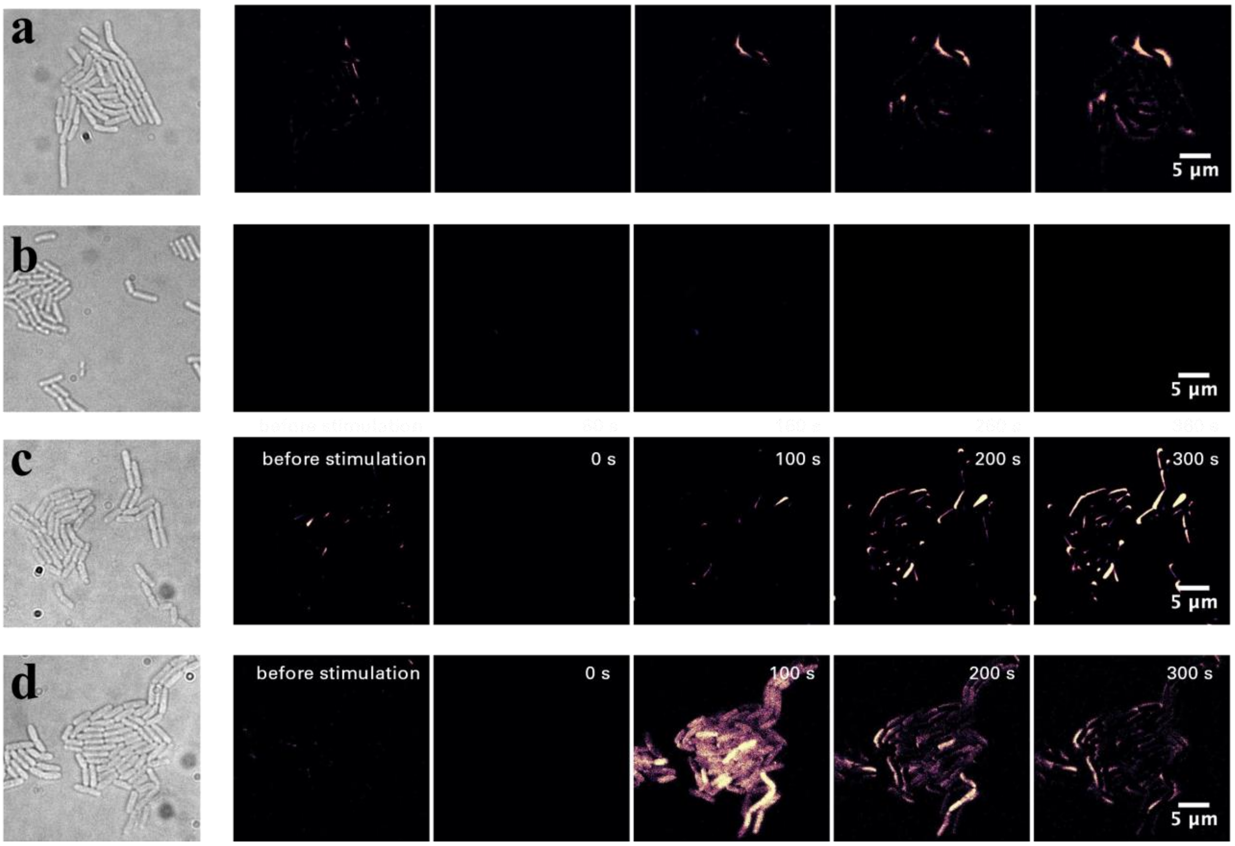

Figure S5. Images of *B. subtilis* membrane potential overtime. **a**) Cell exposed to TMRM only, not stimulated with light – no hyperpolarization observed. **b**) Cell exposed to TMRM and Ziapin 2, not stimulated with light – no hyperpolarization observed. **c**) Cell exposed to TMRM only, stimulated with 470nm light for 10 seconds – no hyperpolarization observed. **d**) Cell exposed to TMRM and Ziapin2, stimulated with 470nm light for 10 seconds – hyperpolarization is observed, followed by depolarization.

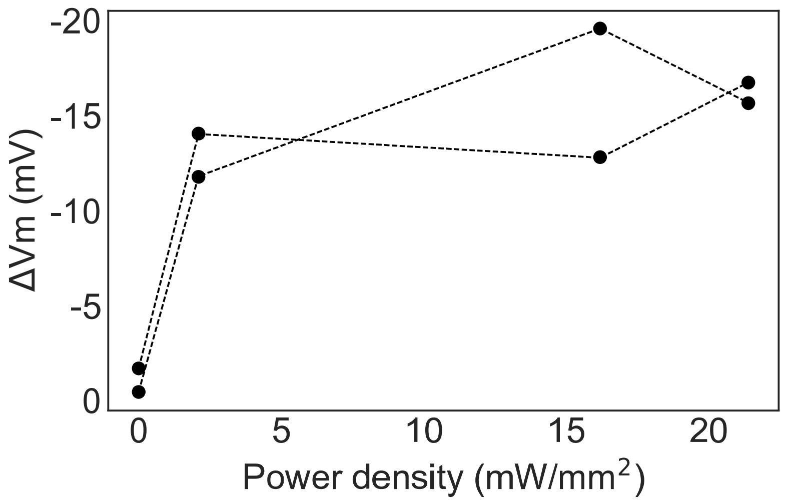

Figure S6. 470 nm light power density of 2 mW/mm^2 is enough to induce Ziapin2-dependent modulation of membrane potential. A peak of induced hyperpolarization is observed with 16 mW/mm^2.

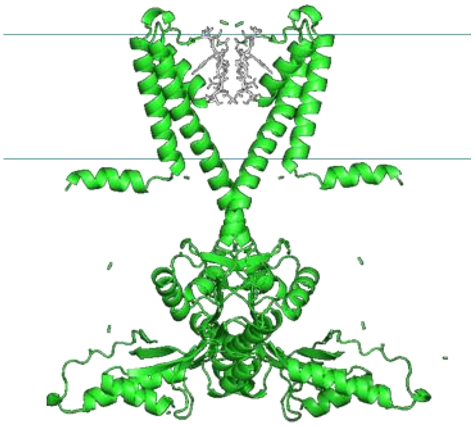

Figure S7 - 3D structure of YugO potassium channel, predicted by alphaFold2 and GalaxyHommer[1,2].

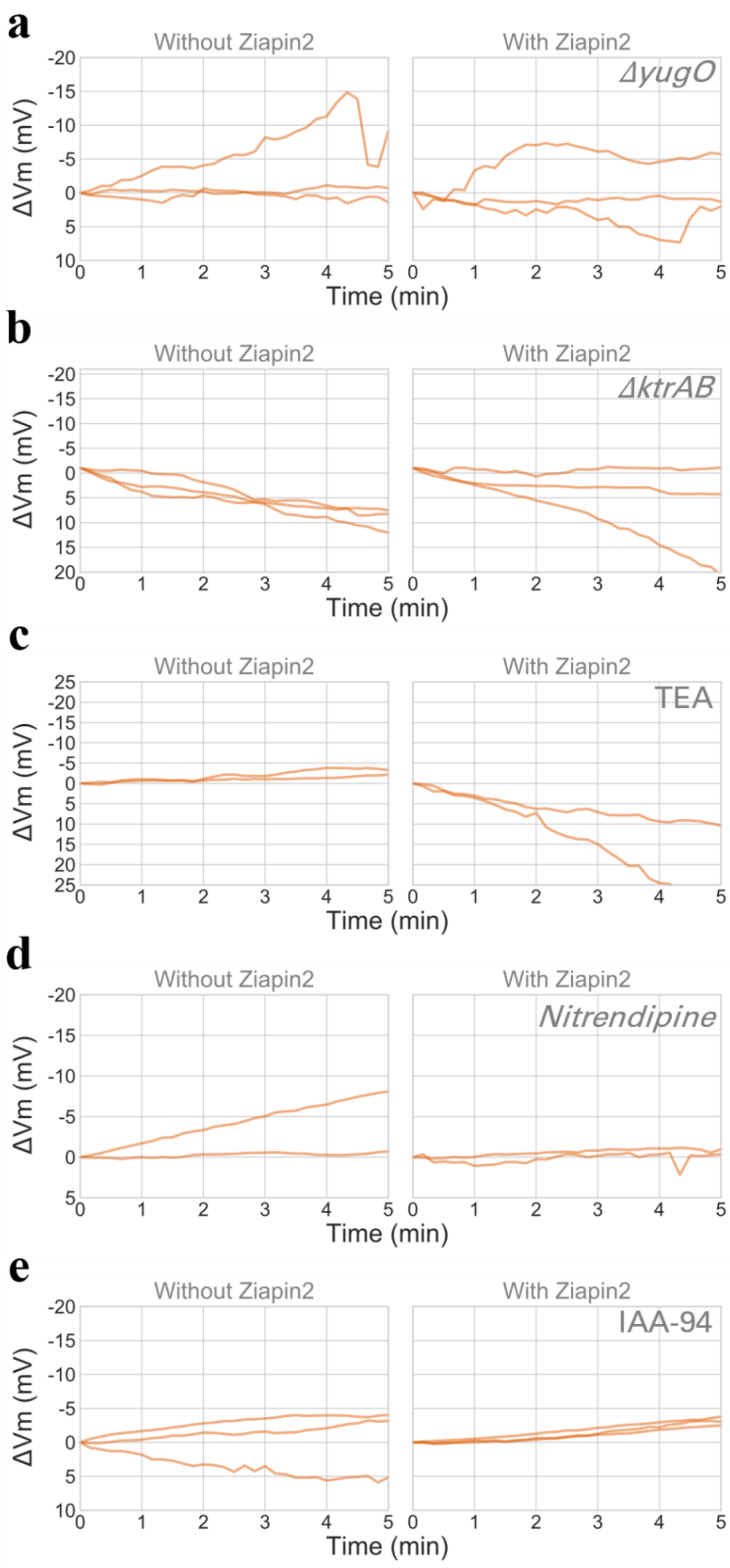

Figure S8 – No 466nm light-stimulation controls. **a**) Δ*yugO* cells with and without Ziapin2. **b**) Δ*ktrAB* cells with and without Ziapin2. **c**) WT cells exposed to TEA, with and without Ziapin2. **d**) WT cells exposed to Nitrendipine, with and without Ziapin2. **d**) WT cells exposed to IAA-94, with and without Ziapin2. Average number of cells analysed per experimental repeat for each condition: >100.

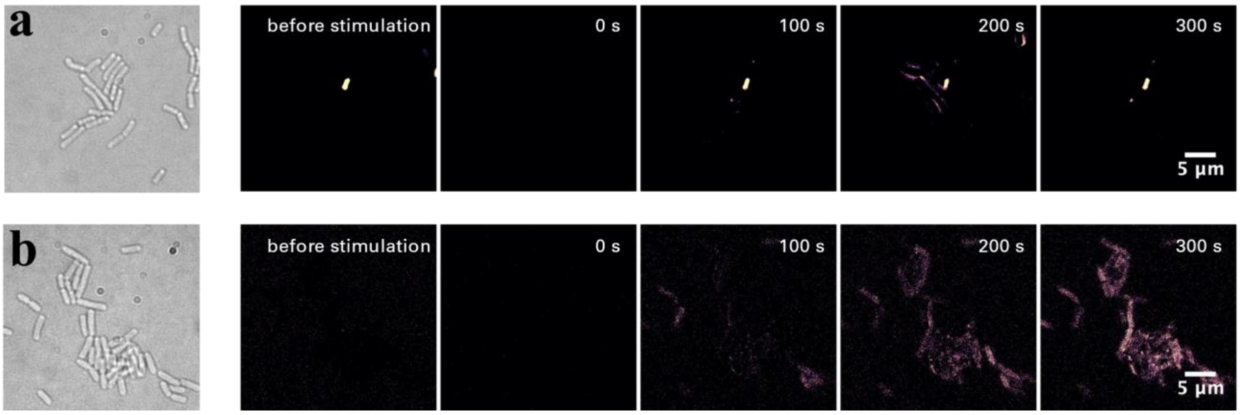

Figure S9. The potassium channel ktrAB and chloride ions are involved in the Ziapin2 induced membrane potential modulation. **a**) ΔktrAB cells exposed to TMRM and Ziapin2 do not show hyperpolarization upon 470 nm light stimulation. **b**) Wildtype cells exposed to TMRM, Ziapin2 and 100 µM of chloride blocker IAA-94 also do not show hyperpolarization upon 470 nm light stimulation.

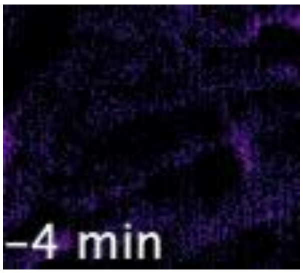

Movie S1. *B. subtilis c*ells exposed to TMRM and Ziapin2. After 470nm light stimulation, cells are hyperpolarized, and gradually depolarize over time.

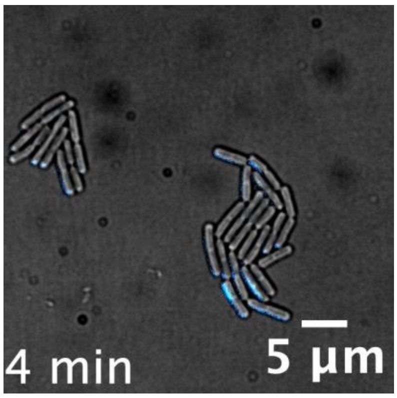

Movie S2. *B. subtilis c*ells exposed to TMRM and Ziapin2, with multiple 470nm light stimulation. Cells hyperpolarized and depolarized multiple times, showing that a oscillatory behaviour can be triggered and controlled by Ziapin2-mediated optomodulation.

Table S1

***B. subtilis* strains**		
**Strain**	**Genotype**	**Source**
NCIB3610	Wildtype	Gift from Süel lab
PY79	Wildtype	Gift from Süel lab
Δ*ktrAB*	ktrAB::neo	Bacillus Genetic Stock Centre (https://bgsc.org/)

Additional Reference

[1] J. Jumper, R. Evans, A. Pritzel, T. Green, M. Figurnov, O. Ronneberger, K. Tunyasuvunakool, R. Bates, A. Žídek, A. Potapenko, A. Bridgland, C. Meyer, S. A. A. Kohl, A. J. Ballard, A. Cowie, B. Romera-Paredes, S. Nikolov, R. Jain, J. Adler, T. Back, S. Petersen, D. Reiman, E. Clancy, M. Zielinski, M. Steinegger, M. Pacholska, T. Berghammer, S. Bodenstein, D. Silver, O. Vinyals, A. W. Senior, K. Kavukcuoglu, P. Kohli, D. Hassabis, *Nature* **2021**, *596*, 583.

[2] M. Baek, T. Park, L. Heo, C. Park, C. Seok, *Nucleic Acids Res.* **2017**, *45*, W320.
